# Determination and prediction of net energy of soybean meal fed to pregnant sows by indirect calorimetry

**DOI:** 10.5713/ab.24.0663

**Published:** 2025-02-27

**Authors:** Lei Xue, Can Zhang, Bo Cheng, Qian Song, Lee J. Johnston, Ling Liu, Fenglai Wang, Jianjun Zang

**Affiliations:** 1 State Key Laboratory of Animal Nutrition and Feeding, College of Animal Science and Technology, China Agricultural University, Beijing, China; 2 West Central Research and Outreach Center, University of Minnesota, Morris, USA

**Keywords:** Net Energy, Prediction, Pregnant Sows, Soybean Meal, Substitution Level

## Abstract

**Objective:**

The study was conducted to investigate the appropriate substitution level of soybean meal (SBM) for determining its net energy (NE), and establish NE prediction equation of SBM based on the determined NE values for pregnant sows.

**Methods:**

In Exp. 1, eighteen pregnant sows (Landrace×Yorkshire; parity, 2 to 3) with an initial body weight (BW) of 221.2±2.6 kg at mid-gestation were blocked by BW and randomly assigned into 3 groups. Three groups fed with a corn-SBM basal diet and two test diets with 15% and 30% energy-supplying components replaced by SBM, respectively. In Exp. 2, six diets were formulated including a corn-SBM basal diet and five SBM diets (based on the substitution level determined of Exp. 1) with different soybean sources and processing methods. Moreover, 12 pregnant pigs (BW = 209.0±3.0 kg; parity, 3 to 4) at mid-gestation were arranged in a 6×3 Youden square design.

**Results:**

Increasing substitution levels of SBM linearly increased (p<0.05) fecal and urinary nitrogen excretion and the ratio of urinary energy to digestible energy (DE), while linearly decreased (p<0.05) the ratio of metabolizable energy (ME) to DE and tended to linearly decrease dietary ME (p = 0.066) and NE (p = 0.074). The coefficient of variation for the NE of SBM was lower at a 15% substitution level compared to a 30% substitution level. The nutritional compositions of SBM are influenced by the soybean sources and processing methods. As dry matter basis, NE values of SBM ranged from 11.1 to 12.7 MJ/kg and the best-fitted prediction equation for NE of SBM was: NE (MJ/kg) = −91.71+5.35×gross energy (%)−0.03×neutral detergent fiber (%; R^2^ = 0.96).

**Conclusion:**

A substitution level of 15% was more appropriate to determine NE of SBM. Furthermore, NE values of SBM can be predicted based on their chemical compositions.

## INTRODUCTION

Soybean meal (SBM) is one of the main protein ingredients in pig feed due to wide availability and excellent amino acid (AA) profile [1,2]. China imports large quantities of soybeans from Brazil and USA every year, which account for almost 76% of the total imported cereals with the increasing demand for soybean products and feed [3]. Much work has been done to improve the utilization of SBM in pigs by evaluating the nutritional values of protein ingredients [4,5]. Cost of feed for sows and their litters represents 15% to 17% of the total feed cost of commercial pig farms [6]. In addition, pregnancy comprises 75% of the entire reproductive cycle of sows during which SBM serves as a vital protein component in their diets. Therefore, accurate evaluation of the available energy in SBM for pregnant sows is of significance for precision nutrition of sows, ultimately leading to reduced feed costs and nitrogen (N) excretion [5].

Net energy (NE) system represents the usable energy of feeds more accurately compared with digestible energy (DE) and metabolizable energy (ME) systems [7]. Recently, a couple of studies have determined NE values of SBM fed to growing-finishing pigs [4,8]. Wang et al [5] used pregnant sows to estimate the DE and ME content of eleven SBM from different sources. However, NE values of SBM fed to pregnant sows have not been reported, since measuring NE is labour-intensive and requires highly specialized equipment and calorimetric trials [9]. The difference method, also known as the substitution method, is used to evaluate the energy value of SBM fed to pigs given the nutritional characteristics of SBM [10]. Furthermore, when using the difference method, the accuracy of NE value was influenced by the level of test ingredients substituting the energy-supplying part in the basal diet [11]. However, the appropriate substitution level of SBM for estimating NE of SBM in pregnant sows remains unclear. Therefore, we conducted research to choose the appropriate substitution level of SBM for determining its NE using the difference method, to determine NE of SBM fed to pregnant sows based on the appropriate substitution level and to establish NE prediction equation for SBM.

## MATERIALS AND METHODS

### Animal care

Animal experiments were carried out in the Fengning Swine Research Unit of China Agricultural University (Chengdejiuyun Agricultural and Livestock Co., Ltd., Hebei, China). The experimental protocols were approved by the Institutional Animal Care and Use Committee of China Agricultural University, Beijing, China (approval number: AW10803202-1-1).

### Sources of soybean meal samples

Six SBM samples (same batch of one SBM sample) were collected from Hebei (n = 1), Henan (n = 2), Heilongjiang (n = 1), and Shandong (n = 2) provinces that included different sources of soybeans, as well as regular and dehulled processes (Table 1). The analyzed chemical compositions of SBM samples are presented in Table 2.

### Animals, dietary treatments and experimental design

In Exp. 1, a total of 18 pregnant sows (Landrace×Yorkshire; parity, 2 to 3; 221.2±2.6 kg at day 60 of gestation) were blocked by body weight (BW) and within each block assigned to one of three dietary treatments in a complete randomized block design comprising of 3 dietary treatments. Each sow was a replicate, and each period contained two sows (i.e., two replicates) for each treatment, with a total of 6 replicates for each treatment in the three periods. Sows were fed three diets (Table 3): a corn-SBM basal diet and two test diets (test diet 15 and test diet 30) with 15% and 30% energy-supplying components replaced by SBM1, respectively. Nutrient concentrations of diets, including standardized ileal digestible indispensable AA, vitamins and minerals, met or exceeded nutritional requirements of pregnant sows with 2 to 4 parities and less than 90 days of gestation [1].

In Exp. 2, twelve pregnant sows (Landrace×Yorkshire; parity, 3 to 4; initial BW 209.0±3.0 kg at day 41 of gestation) were assigned to a replicated 6×3 Youden square design with six diets and three consecutive periods in each square. Each sow was a replicate, and each period contained 1 sow (i.e., 1 replicate) for each treatment, with a total of 6 replicates for each treatment in the six periods. Experimental diets included six diets (Table 4), a corn-SBM basal diet and 5 SBM diets in which the test SBM replaced 15% of corn and SBM in the basal diet. A substitution level of test SBM was selected based on results of Exp. 1. Dietary nutrients met or exceeded the nutritional requirements of pregnant sows with 3 to 4 parities and less than 90 days of gestation [1].

### Experimental management

According to a previous report [12], each period lasted for 10 days, including a 5-day period for dietary adaptation, a 1-day adaptation of the chambers, a 3-day heat production (HP) measurement period, and 1-day measurement of fasted heat production (FHP). Sows were fed in metabolic cages (1.7 m×0.7 m×1.4 m) during the adaptation period and transferred to open-circuit respiratory calorimetric chambers during the formal trial period [13]. The feed intake was restricted to 1.5×the ME required for maintenance (150 kcal ME/kg BW^0.75^/day) based on the initial BW of sows on day 0 [14]. The feeding level in each trial period was adjusted to maintain BW and backfat thickness of sows constant throughout the experiments [12]. Sows were fed equal quantities twice daily (08:30 and 15:30 h) and allowed ad libitum access to water. The open-circuit respiratory calorimetric chambers were cleaned every morning to ensure a clean and hygienic environment. During the calorimetric trial, temperature and relative humidity of the chambers were maintained at 20±1°C and 75±5%, respectively. The wind speed and illumination time in the chambers were 0.5 m/s and 12 h (from 06:00 to 18:00 h), respectively. Before the HP measurement, the ethanol combustion test results showed that the recovery rates of CO_2_ and recovery factors of O_2_ met the requirements of indirect calorimetry [15]. The sensors and gas concentrations were calibrated according to the description of Wang et al [13].

### Sample collection and preparation

Six SBM samples were collected after crushing SBM ingredients by the feed grinder, while dietary samples were collected during preparation. From days 7 to 9, feces were collected using a time-based collection method and urine samples were accomplished. Gas exchange (including O_2_, CO_2_ and CH_4_) was measured according to procedures reported by Wang et al [13]. HP was measured using indirect calorimetry through gas exchanges nearly 22 h after deducting the time of feeding, collecting samples and adding water. Gas exchange data from 10:30 h (day 10) to 06:30 h (day 11) of each period were used to calculate FHP, while urine samples were collected separately. Before the collection of urinary samples, 300 mL of 10% hydrochloric acid was added to each urine collector vessel to prevent N volatilization. Urine was mixed, filtered with 8 layers of gauze and 2% of total urine volume was sampled and retained. Fecal and urinary samples were stored at −20°C. At the time of measuring FHP, urine was collected separately. Feces were baked at 72°C for 72 h and moisture was regained at ordinary temperature for 24 h before fecal samples were thawed and fully mixed. The crushed SBM, diets and feces were sifted through a 40-mesh seize and bagged for analysis [13].

### Chemical analysis

Samples of feces, diets and SBM collected in both experiments were analyzed for dry matter (DM, method 934.01), crude protein (CP; method 984.13), ether extract (EE; method 920.39), ash (method 942.05), calcium (Ca; method 927.02), phosphorus (P; method 965.17) and AA (method 928.30) following methods described by AOAC International [16]. An earlier method reported by Johansen et al [17] was used to determine the concentrations of three oligosaccharides (sucrose, raffinose and stachyose) in SBM. Gross energy (GE) of SBM, diets, feces and urine was measured by an isoperibolic calorimeter (Parr 6400; Parr Instrument, Moline, IL, USA). Concentrations of neutral detergent fiber (NDF) and acid detergent fiber (ADF) were determined by a fiber analyzer (Ankom 200 Fiber Analyzer; Ankom Tecnnology, Macedon, NY, USA) according to Van Soest et al [18]. Amino acids of SBM samples and diets were analyzed using an AA analyzer (Hitachi L-8900; Hitachi Ltd., Tokyo, Japan) and high-performance liquid chromatography (Agilent 1200 Series; Agilent Technologies Inc., Santa Clara, CA, USA) according to the earlier method [16].

### Calculation

The FHP and HP were calculated using the following equation reported by Brouwer [19]:


$$\text{HP }(\text{kJ})=16.1753\times {\text{O}}_{2}\;(\text{L})+5.0208\times {\text{CO}}_{2}(\text{L})-2.1673\times {\text{CH}}_{4}(\text{L})-5.9873\;\text{urinary N }(\text{g})$$

where HP was the mean value of HP over 3 days and L was the abbreviation of liter. The FHP was calculated using the same equation for HP.

The retained dietary energy (RE) and NE were calculated according to Noblet et al [20] using the following equations:


$$\begin{array}{l} \text{RE }(\text{kJ}/\text{kg DM})=[\text{ME intake }(\text{kJ}/\text{day})-\text{HP }(\text{kJ}/\text{day})]/[\text{dry matter intake }(\text{DMI})\;(\text{kg}/\text{day})]\\ \text{NE }(\text{kJ}/\text{kg DM})=[\text{RE }(\text{kJ}/\text{day})+\text{FHP }(\text{kJ}/\text{day})]/\text{DMI }(\text{kg}/\text{day}) \end{array}$$

The DE and ME of diets, DE of SBM and the apparent total tract digestibility (ATTD) of GE of diets were calculated using the following the equations described by Adeola [10]:


$$\begin{array}{l} {\text{DE}}_{\text{diet}}\;(\text{MJ}/\text{kg})=[{\text{GE}}_{\text{diet}}\;(\text{MJ})-{\text{GE}}_{\text{feces}}(\text{MJ})]/\text{DMI }(\text{kg})\\ {\text{ME}}_{\text{diet}}\;(\text{MJ}/\text{kg})=[{\text{GE}}_{\text{diet}}\;(\text{MJ})-{\text{GE}}_{\text{feces}}(\text{MJ})-\text{urinary energy }(\text{UE};\text{MJ})-{\text{CH}}_{4}\text{E }(\text{MJ})]/\text{DMI }(\text{kg})\\ {\text{DE}}_{\text{SBM}}\;(\text{MJ}/\text{kg})=[{\text{DE}}_{\text{SBM diet}}\;(\text{MJ}/\text{kg})-{\text{DE}}_{\text{basal diet}}(\text{MJ}/\text{kg})\times (1-\text{X})]/\text{X}/0.97\\ \text{ATTD of GE}=[{\text{GE}}_{\text{intake}}(\text{MJ})-{\text{GE}}_{\text{feces}}(\text{MJ})]/{\text{GE}}_{\text{intake}}(\text{MJ}) \end{array}$$

where DE and ME were calculated based on DM. “X” represents the level of test SBM substituting energy-supplying components of the corn-SBM basal diet. The ME and NE of SBM were calculated using the same equation for the DE of SBM. The ATTD of DM, CP and, organism (OM) were also calculated using the same equation for the ATTD of GE.

The retained N and N net utilization were calculated using the following equation reported by Wang et al [5]:


$$\begin{array}{l} \text{Retained N }(\text{g})=\text{N intake }(\text{g})-\text{Fecal N }(\text{g})-\text{Urinayr N }(\text{g})\\ \text{N net utilization }(\%)=100\times [\text{Retained N }(\text{g})/\text{N intake }(\text{g})] \end{array}$$

The retained energy as protein (RE_P_) was calculated based on the retained N. The retained energy as lipids (RE_L_) was calculated as the difference between RE and RE_P_ [21]. Respiratory quotient (RQ) was defined as the ratio of CO_2_ production to O_2_ consumption over the same time period. The RE_P_, RE_L_ and RQ were calculated using the following equations:


$$\begin{array}{l} {\text{RE}}_{\text{P}}\;(\text{kJ}/\text{day})=\text{Retained N }(\text{g}/\text{day})\times 6.25\times 23.86\;(\text{kJ}/\text{g})\\ {\text{RE}}_{\text{L}}\;(\text{kJ}/\text{day})=\text{RE }(\text{kJ}/\text{day})-{\text{RE}}_{\text{P}}\;(\text{kJ}/\text{day})\\ \text{RQ}={\text{CO}}_{\text{2}}\;(\text{L}/\text{d})/{\text{O}}_{2}\;(\text{L}/\text{d}) \end{array}$$

### Statistical analysis

The MIXED procedure of SAS 9.4 (SAS Institute Inc., Cary, NC, USA) was used to analyze all data. Each pregnant sow was considered as an experimental unit in the study. In the statistical model, diet or SBM was seen as fixed effects, while replication, chamber, period and sows within replication were considered as random effects. The mean of each treatment was calculated by the LSMEANS statement of SAS. The linear and quadratic effects of increasing substitution levels of SBM in Exp. 1 were tested by polynomial contrasts. Tukey’s multiple range test was used to examine the significance of differences among different SBM treatments in Exp. 2. A probability of p<0.05 was considered significant, whereas p<0.10 was considered a tendency. Correlation coefficients between NE values and chemical compositions were determined by the CORR procedure of SAS. The stepwise regression was used to select significant variables and establish the prediction equations for DE, ME and NE of SBM based on its chemical composition. The R^2^, root mean square error and p-value were used as indicators to evaluate the best-fitted prediction equations.

## RESULTS

### Chemical compositions of soybean meals and diets

As presented in Table 2, the coefficients of variation (CV) of EE, NDF, ADF, sucrose, raffinose, and stachyose of 6 SBM samples were greater than 10%, while the CVs of 18 AA were less than 10%. Based on the DM, the contents of Lys, Met, Trp and Thr in SBM ranged from 2.87% to 3.20%, 0.59% to 0.75%, 0.63% to 0.72% and 1.80% to 1.95% with averages of 3.08%, 0.69%, 0.69% and 1.88%, respectively. The highest CP concentration in SBM was 53.03% (SBM3), and the lowest was 48.96% (SBM1).

From Table 3, the SBM1 contents of the 2 test diets were different. Test diet 30 showed higher CP, NDF, ADF, Lys and Met contents compared with test diet 15. Five SBM diets presented similar chemical compositions (Table 4).

### Nutrients digestibility, energy utilization and N balance of diets

Nutrients digestibility, energy utilization and N balance of diets in Exp. 1 are shown in Table 5. Increasing substitution level of SBM linearly and quadratically improved (p<0.05) dietary ATTD of CP. Dietary CH_4_E/DE ratio was not affected by the substitution level of SBM. The N intake, fecal N output, urinary N output and UE/DE of diets were linearly increased (p<0.05) with increasing substitution level of SBM. In addition, a linear decrease (p<0.01) of ME/DE ratio of diets was observed with increasing substitution level of SBM. Increasing substitution level of SBM did not affect the N net utilization of diets.

As shown in Table 6, the corn-SBM basal diet showed higher ATTD of DM (p<0.05) and OM (p<0.05) in comparison with SBM diet 3, SBM diet 4 and SBM diet 6. In terms of energy utilization, no differences were observed in the UE/DE, CH_4_E/DE or ME/DE ratios across the six treatments. Sows fed SBM diets presented greater (p<0.05) N intake in comparison with those fed the basal diets. Compared with other diets, SBM diet 3 showed a tendency (p = 0.074) of increased urinary N output. Nitrogen retained in SBM diets (except for SBM diet 3) tended (p = 0.053) to be higher than that in basal diets. Interestingly, fecal N output and N net utilization were not different among the six dietary treatments.

### Effects of dietary treatments on energy balance of pregnant sows and energy values of diets

Effects of dietary treatments on the energy balance of pregnant sows and energy values of diets in Exp. 1 are listed in Table 7. The ME intake was linearly decreased (p<0.05) with the increase of substitution level of SBM. There was a tendency for a quadratic decrease (p = 0.075) in the THP as the substitution level of SBM increased. Increasing substitution level of SBM linearly and quadratic decreased (p<0.05) FHP of sows. Increasing dietary substitution level of SBM linearly improved (p<0.05) RE_P_ of pregnant sows. The RE and RE_L_ of sows were not affected by the substitution level of SBM. Dietary ME (p = 0.066) and NE (p = 0.074) tended to linearly decrease with increasing substitution level of SBM. Increasing substitution level of SBM did not affect DE of diets.

The ME intake, THP, FHP RE_L_, and RE were not different among the five SBM diets (Table 8). Compared with sows fed the basal diet, sows fed SBM diets (except for SBM diet 3) presented higher (p<0.05) RE_P_. Among the five SBM diets, the RE of pregnant sows in the three dehulled SBM diets (SBM diet 2, 3 and 5) exceeded 100.0 kJ/kg BW^0.75^/day. No significant differences in RQ, DE, ME and NE were observed among the six treatments.

### Available energy values and energy utilization of soybean meals

The DE, ME, NE, ratios of ME/DE and NE/ME of SBM were not significantly different between the substitution levels of 15% and 30% in Exp. 1 (Table 9). The average NE contents in SBM with 15% and 30% substitution levels were 11.9 and 11.4 MJ/kg DM, respectively. Upon further calculations, it was observed that the CV of NE was lower at a substitution level of 15% (12.1%) compared that at a substitution level of 30% (13.5%).

The available energy values and energy utilization of five SBM samples are presented in Table 10. No significant differences were observed in DE, ME, NE, ME/DE and NE/ME among the 5 SBM samples. The numerical differences between the maximum and minimum values of DE, ME and NE were 2.5, 2.6 and 1.6 MJ/kg DM, respectively. The average NE values of three dehulled SBM samples and two regular SBM samples were 12.6 and 11.4 MJ/kg DM, respectively.

### Correlation and prediction equations for net energy of soybean meals for pregnant sows

Correlation coefficients among chemical compositions and available energy values of the 6 SBM samples were based on results of Exp. 1 and Exp. 2 (Table 11). The GE content was correlated positively with NE (r = 0.96, p<0.01), and ME displayed a positive correlation with DE (r = 0.98, p<0.01) for pregnant sows.

Prediction equations of available energy in SBM for pregnant sows are presented in Table 12. The DE in SBM for pregnant sows can be predicted using its GE and ash. Furthermore, the ME can be estimated by its DE. Additionally, GE and NDF served as the optimal predictors of NE in SBM.

## DISCUSSION

### Chemical compositions of soybean meals and diets

Based on the known nutrient contents, six SBM samples with high variation in chemical composition were selected from Chinese feed enterprises. The SBM were processed with different sources of soybeans, as well as regular and dehulled processing technology. The EE content in SBM was relatively low, whereas the CV of EE was within the range reported by others [2,5]. According to Grieshop et al [22], the method of solvent extraction extracted approximately 99% of the oil from soybeans, resulting in a relatively low EE content in SBM. The contents of other components, including GE, NDF, ADF and 18 AA, of the six SBM samples were close to values reported [1]. Chemical characteristics of SBM are affected by many factors, such as soybean variety, growth environment, and processing methods [2,5]. Moreover, the difference between regular and dehulled SBM lies in whether soybean hulls are reintroduced into the meal or not [2]. Consequently, processing methods led to differences in chemical composition between regular and dehulled SBM. In agreement with Li et al [2], SBM from Brazil contained more CP than those from China and the United States.

A corn-SBM basal diet is more suitable than a diet based solely on corn for the determination of the available values due to a more favorable balance of nutrients [16]. Hence, both experiments in the study used corn-SBM basal diets. According to Villamide [23], increasing the substitution level of the test ingredients in diets can improve the precision of energy values. However, high levels of test ingredients may increase the difference of chemical composition between test and basal diets, which may interfere with the use of the basal diet and lead to erroneous estimation of the energy values of the ingredients [23]. Because of different substitution level of SBM in the first experiment, CP, NDF, ADF, Lys and Met contents of two test diets of were different in Exp. 1, which was in agreement with Kim et al [11] (different substitution level of canola meal). This may be primarily attributed to the different content between SBM and corn of diets, coupled with their distinct chemical compositions [1]. In order to reduce the interaction effect of AA, no additional amino acids were added to the experimental diets to balance AA [24]. All SBM diets contained identical proportions of SBM and corn, resulting in comparable chemical compositions across the 5 SBM diets in Exp. 2, which was in line with previous research [2,5].

### Nutrients digestibility, energy utilization and N balance of diets

Dietary CP content displayed a positive effect on ATTD of CP [25]. The ATTD of CP increased with increasing dietary CP levels in Exp. 1, which may be due to a decrease in the fecal N excretion as a percentage of N intake in this study. The ATTD of CP is calculated as the ratio of CP intake minus fecal CP excretion to CP intake, whereas fecal CP included undigested CP excretion from diet and the basal endogenous losses of CP. As CP intake increases, the ratio of basal endogenous losses of CP to CP intake decreased, thereby increasing the ATTD of CP. This observation is consistent with the results of Kim et al [26]. Therefore, increasing substitution level of SBM improved dietary ATTD of CP, which may be related to higher dietary CP contents in Exp. 1. No differences of ATTD of DM, GE, CP and OM were observed among five SBM diets in Exp. 2. This may be related to the similar nutritional compositions of diets [12]. Likewise, 11 SBM diets with similar nutrients presented the similar ATTD of GE in mid-gestation and late-gestation sows [5].

Increasing substitution level of SBM increased dietary UE/DE ratio of diets, while decreased dietary ME/DE ratio in Exp. 1. The possible reason was that the excessive protein intake reduced the utilization of energy [25]. Kim et al [26] showed that dietary UE and N increased with increasing dietary CP levels. According to a previous report [27], each gram of total intestinal digestible CP more than needed increased the loss of UE by approximately 3 kJ after deamination. Therefore, test diet 30 showed a higher UE loss than test diet 15 due to urinary N output in Exp. 1, which was in agreement with Le Goff and Noblet [25], who showed that the higher N excretion with high CP diets was accompanied by the increased UE loss. In addition, the ME/DE ratio of 5 SBM diets was 95.3% in Exp. 2, which was lower than 96.4% of the 5 fiber-rich diets [13]. This may be due to the increased UE/DE ratio caused by high dietary CP intake [25]. Interestingly, the CH_4_E/DE ratio of 5 SBM diets (1.0%) was close to 5 fiber-rich diets (1.1%), although the average dietary fiber of SBM diets in our study (9.2%) was lower in contrast to the findings of Wang et al [13] at 24.4%. In addition, Le Goff et al [28] demonstrated that the utilization of dietary energy in adult sows is little affected by the addition and origin of dietary fiber.

The fecal N output and urinary N output were increased with increasing substitution level of SBM in Exp. 1, which may be attributed to a higher CP intake [25,26]. However, increasing substitution level of SBM did not affect N net utilization of diets in Exp.1. A possible reason for this could be that N net utilization of sows was not improved with an increase of the dietary CP levels [29], even when these values exceeded to the recommendations of the NRC [1]. Le Bellego et al [24] indicated that dietary CP levels and the balance of AA influenced N excretion and utilization of ME. There were no significant differences in N intake, N excretion, retained or net utilization among the five SBM diets in Exp. 2, as the CP levels of these diets were close. Endogenous secretion of N in pigs could not be influenced by dietary CP content, and only a small amount of N is discharged in the form of fecal N after taking N-containing diets [30]. However, further studies are necessary to elucidate the mechanism of N net utilization.

### Effects of dietary treatments on energy balance of pregnant sows and energy values of diets

During adaptation from the fed state to the fasted state, pigs were fed at energy intake levels near maintenance [9,14]. Increasing substitution level of SBM tended to quadratically decrease THP of pregnant sows in Exp. 1. This may be related to ME intake and nutrient oxidation [29]. The FHP was not affected by different SBM diets in Exp. 2. Li et al [8] and Pérez de Nanclares et al [29] demonstrated that FHP of pigs was not influenced by dietary CP or EE despite exceeding the recommendations of the NRC [1]. In addition, the average THP of pregnant sows fed five SBM diets was 453.0 kJ/kg BW^0.75^/day in Exp. 1, which was slightly higher than the earlier values [13,25,31]. According to Ramonet et al [31] and Wang et al [13], the THP is affected by various factors, including the physiological state of sows (pregnant and nonpregnant), dietary composition and feeding levels. Furthermore, we speculated that the elevated HP observed in this research may also be attributed to the lack of satiety, caused by restricted feeding practices and the rapid digestion of corn-SBM diets [32]. Subsequently, compared with sows fed fiber-rich diets, those fed corn-SBM diets exhibited increased standing time and HP. Unfortunately, HP was not further subdivided according to the experimental conditions in this study.

The RE_P_, RE_L_ and RE are affected by ME and types of diets [13], and also are associated with parity and state of pregnancy [33]. Beyer et al [33] illustrated that the RE_P_ and RE in pregnant sows exhibited exponential functional growth as time progresses. Pregnant sows that were fed two test diets in Exp. 1 and five SBM diets in Exp. 2 exhibited comparable RE_P_, RE_L_ and RE, potentially due to similar physiological conditions and feed intake. The energy extracted from diets of sows was distributed in priority to maintenance, growth of conceptus, RE_P_, and RE_L_ needs [34]. When energy intake was insufficient, lipid provided the needed energy by lipolysis. Wang et al [13] determined NE content of five fiber-rich ingredients fed to pregnant sows, and reported the RE_L_ of five diets with fiber-rich ingredients ranged from 14 to 127 kJ/kg BW^0.75^/day. The RE_L_ varied greatly, which was in agreement with our study of five SBM diets in Exp. 2. Meanwhile, the RE_L_ of pregnant sows fed diets with SBM6 was less than 0, which may be caused by inadequate energy intake [34].

Dietary DE was not affected by the substitution level of SBM in Exp. 1. The DE contents in diets were associated with dietary GE contents and ATTD of GE, while ME was directly related to DE, UE and CH_4_E [13]. Increasing substitution levels of SBM tended to linearly decrease dietary ME and NE in Exp. 1. This may be due to the increased UE excretion [26] and higher thermic effect of food associated with high protein intake as opposed to low protein diets. The SBM diets exhibited comparable DE and NE values in Exp. 2, aligning with previous research findings [2,5,25].

### Available energy values and energy utilization of soybean meals

The available energy presented in plant-based ingredients is influenced by individual animals, plant growing conditions, and processing and determination methods [5,28,34]. In the current study, there were no significant differences in available energy values and energy utilization of SBM between 15% and 30% energy-supplying components replaced by SBM in Exp. 1. However, based on further calculation, it was observed that the CV of NE in SBM with a substitution level of 15% was less than that with a substitution level of 30% in Exp. 1. This suggests that the data measured at a 15% substitution level exhibited greater stability and repeatability [35]. Huang et al [36] demonstrated that the CV of DE content of the inclusion level of 28.8% wheat middling was slightly (about 0.8%) lower than inclusion levels of 38.4% wheat middling. Compared with this, the difference between the inclusion level of SBM in the current study was greater (15% and 30%). The substitution level closer to the actual supplementation level in pig production is more appropriate for determining the energy values of ingredients [36]. Compared with the substitution level of 30%, the substitution level of 15% of SBM is more close to the actual supplementation level of pregnant sows diets in this study. In general, taking into account N balance, energy utilization of diets, numerical stability of results and actual supplementation level, it was concluded that a substitution level of 15% of SBM was more appropriate for determining NE values of SBM in pregnant sows by the difference method.

Consistent with previous reports [2], the available energy values of SBM samples derived from various soybean sources and processed by different methods varied greatly. Given this scenario, accurate evaluation of SBM nutritive values is of great significance for precise nutrition [5]. Compared to regular SBM, dehulled SBM had higher DE and ME values in Exp. 2, which was in agreement with previously reported data [5]. Clearly, the present study shows that the average DE, ME and NE values of three dehulled SBM samples in Exp. 2 exceed those documented by the NRC [1]. This can be attributed to the different physiological stages of pigs used in experiments. In the current study, pregnant sows were used as experimental animals, while NRC [1] compiled more data related to growing pigs. Moreover, variation in the nutritional compositions of SBM can also result in different available energy values. For example, total contents of three oligosaccharides (sucrose, raffinose and stachyose) of dehulled SBM reported by the NRC [1] were higher than those determined of three dehulled SBM in the study. These oligosaccharides cannot be digested effectively by pigs due to lack of α-galactosidases, but can be used by bacteria in large intestine of pigs [37]. This may cause flatulence, thereby leading to a decrease in the ATTD of nutrients [37]. The ME/DE ratio of SBM in Exp. 2 in the current study was less than the reported values [2,5]. This may be attributed to the deduction of methane energy, which can be obtained by indirect calorimetry [13]. Moreover, this is related to different physiological stage and nutritional levels of basal diets. Adult sows showed lower N retention and higher urinary energy loss than growing pigs [25]. Likewise, the ME/DE ratio of canola meal in corn-SBM basal diets was lower than that in corn basal diets [11]. Furthermore, due to the influence of pregnancy duration and the number of chambers, the adaptation and collection periods were found to be shorter than those reported by Wang et al [5], while remaining within the standard range for energy measurement [9,12].

### Correlation and prediction equations for net energy of soybean meals for pregnant sows

The DE was positively correlated with ME of SBM for pregnant sows, and comparable finding was reported by Wang et al [5]. Additionally, NE displayed a positive correlation with GE of protein ingredients, which was in line with Li et al [4]. The NE values obtained from several SBM samples cannot represent the NE of all SBM utilized in the feed industry. According to Zhang et al [7] and Święch [38], a prediction equation based on chemical composition provides a relatively convenient and rapid approach for estimating the NE of ingredients. The intestinal tract of adult sows is more developed than that of growing pigs, resulting in higher digestibility of nutrients [28]. Therefore, the DE, ME and NE values may be underestimated when the linear prediction equations of SBM for growing pigs are used for pregnant sows [2,14,38]. In agreement with Noblet and Perez [39], GE was utilized as a predictor for the DE and ME regression equations. Additionally, the DE of SBM was employed to predict ME values [2,5], whereas GE and NDF were considered predictors for NE prediction equations of protein ingredients [4,20]. Using the chemical compositions of SBM outlined in the Nutrient Requirement of Swine in China [40], and applying the prediction for NE values of SBM, the estimated NE values for dehulled and regular SBM fed to pregnant sows were 12.6 and 12.1 MJ/kg DM, respectively. Notably, these predicted NE values surpass the reported values (11.3 and 11.2 MJ/kg DM) [40]. In addition, the average NE of dehulled and regular SBM were 12.4 and 11.4 MJ/kg DM, respectively, if the chemical compositions of 6 SBM samples in the study were used to predict the NE of SBM by the equation. Similarly, the values were higher than the documented values by NRC [1], while these were close to the measured average NE of dehulled and regular SBM (12.6 MJ/kg DM and 11.5 MJ/kg DM) in the study. Nevertheless, due to the limited number of SBM samples, there is a need to enhance the prediction equations for NE of SBM fed to pregnant sows, and the accuracy of the equation demands further validation.

## CONCLUSION

In summary, the chemical characteristics of SBM are influenced by both the soybean sources and the processing methods, particularly in terms of the EE, NDF, ADF and oligosaccharides. When determining the NE in SBM of pregnant sows by using the difference method, a substituting 15% of the energy-supplying ingredients with SBM enhances the accuracy of the estimates. Furthermore, the GE and NDF content of SBM can serve as the predictors of NE values in SBM for pregnant sows.

## Figures and Tables

**Table 1:** Source information of 6 soybean meal (SBM) samples^1)^

Sample number	Location of processing enterprises in China	Soybean source	Dehulled /Regular^2)^
SBM1	Hebei	USA	Regular
SBM2	Heilongjiang	China	Dehulled
SBM3	Shandong	Brazil	Dehulled
SBM4	Shandong	Brazil	Regular
SBM5	Henan	USA	Dehulled
SBM6	Henan	USA	Regular

1) All soybean meal samples were solvent-extracted.

2) Dehulled means that no hulls were added after crushing and Regular means that soybean hulls were added to the crushed meal.

**Table 2:** Nutrient composition of soybean meal (SBM) samples (%, DM basis)

Nutrient	SBM1	SBM2	SBM3	SBM4	SBM5	SBM6	Mean	CV
DM	89.29	90.30	89.73	90.70	89.51	89.88	89.90	0.58
GE (MJ/kg)	19.47	19.55	19.55	19.36	19.48	19.28	19.45	0.56
CP	48.96	50.40	53.03	49.26	51.53	50.45	50.61	2.97
EE	0.77	1.34	0.57	1.09	0.71	0.75	0.87	32.82
NDF	21.70	9.04	11.59	15.58	10.52	11.32	13.29	35.04
ADF	7.87	4.63	6.07	9.01	5.62	6.41	6.60	24.03
Ash	6.94	7.09	7.22	6.79	6.76	6.63	6.91	3.20
Ca	0.33	0.32	0.34	0.33	0.32	0.31	0.33	3.23
P	0.69	0.81	0.71	0.66	0.70	0.68	0.71	7.44
Oligosaccharide								
Sucrose	7.39	8.41	6.38	5.87	7.21	7.31	7.10	12.43
Raffinose	0.74	0.80	1.44	1.29	0.95	1.17	1.07	26.27
Stachyose	4.29	6.04	4.33	3.96	5.90	5.68	5.03	18.60
Indispensable AA								
Arg	3.19	3.32	3.37	2.98	3.34	3.24	3.24	4.05
His	1.21	1.23	1.26	1.17	1.23	1.23	1.22	2.24
Ile	2.15	2.08	2.29	2.10	2.21	2.16	2.17	3.23
Leu	3.51	3.44	3.76	3.45	3.64	3.59	3.57	3.16
Lys	3.20	3.08	3.11	2.87	3.13	3.06	3.08	3.31
Met	0.59	0.74	0.75	0.67	0.70	0.71	0.69	7.66
Phe	2.31	2.46	2.67	2.44	2.58	2.59	2.51	4.73
Thr	1.90	1.84	1.95	1.80	1.93	1.88	1.88	2.72
Trp	0.69	0.70	0.69	0.63	0.72	0.72	0.69	4.37
Val	2.27	2.18	2.36	2.13	2.29	2.23	2.24	3.33
Dispensable AA								
Ala	2.08	2.31	2.45	2.27	2.35	2.31	2.30	4.85
Asp	5.33	5.01	5.45	4.96	5.32	5.16	5.21	3.41
Cys	0.68	0.78	0.70	0.65	0.70	0.68	0.70	5.75
Glu	8.51	8.16	9.01	8.17	8.91	8.70	8.58	3.86
Gly	2.02	1.91	2.05	1.90	1.99	1.97	1.97	2.75
Pro	2.34	2.27	2.37	2.16	2.37	2.37	2.31	3.34
Ser	2.34	2.18	2.37	2.11	2.35	2.30	2.28	4.24
Tyr	1.43	1.72	1.86	1.70	1.79	1.75	1.71	7.89

CV, coefficient of variation; DM, dry matter; GE, gross energy; CP, crude protein; EE, ether extract; NDF, neutral detergent fiber; ADF, acid detergent fiber; Ca, calcium; P, total phosphorus; AA, amino acid; Arg, arginine; His, histidine; Ile, isoleucine; Leu, leucine; Lys, lysine; Met, methionine; Phe, phenylalanine; Thr, threonine; Trp, tryptophane; Val, valine; Ala, alanine; Asp, aspartic acid; Cys, cysteine; Glu, glutamic acid; Gly, glycine; Pro, proline; Ser, serine; Tyr, tyrosine.

**Table 3:** Formulation and nutritional content of diets in Exp. 1 (%, as-fed basis)

Ingredient	Basal diet	Test diet 15	Test diet 30
Corn	90.00	76.50	63.00
SBM	7.00	5.95	4.90
SBM1	-	14.55	29.10
Dicalcium phosphate	1.30	1.30	1.30
Salt	0.80	0.80	0.80
Limestone	0.40	0.40	0.40
Premix^1)^	0.50	0.50	0.50
Nutrient levels^2)^			
GE (MJ/kg)	15.93	16.12	16.29
CP	10.48	15.89	20.63
EE	1.48	1.32	1.17
NDF	7.96	9.30	9.52
ADF	2.06	3.00	3.56
Ash	3.88	3.66	3.92
Ca	0.66	0.86	0.73
P	0.49	0.54	0.54
Lys	0.49	0.79	1.13
Met	0.15	0.19	0.27

1) Premix provided per kilogram of complete feed: 6,000 IU of vitamin A (as retinyl acetatel), 3,000 IU of vitamin D_3_ (as cholecalciferol), 20 IU of vitamin E (as dl-alpha-tocopheryl acetate), 1.8 mg of vitamin K_3_ (as menadione nicotinamide bisulfite), 2.0 mg of vitamin B_1_ (as thiamine), 6.0 mg of vitamin B_2_ (as riboflavin), 4.0 mg of vitamin B_6_ (as pyridoxine hydrochloride), 3,000 mg of choline, 0.02 mg of vitamin B_12_ (as cobalamin), 26.0 mg of niacin, 18.0 mg of pantothenic acid (as dl-calcium pantothenate), 3.2 mg of folic acid, 0.4 mg of biotin, 400 mg of Fe (as FeSO_4_·H_2_ O), 20 mg of Cu (as CuSO_4_·5H_2_O), 100 mg of Zn (as ZnO), 50 mg of Mn (as MnO), 1.2 mg of I (as KI), 0.30 mg of Se (as Na_2_ SeO_3_), 8.0 g of Ca (as calcium pantothenate), 0.8 g of P (as calcium hydrogen phosphate), 5.6 g of sodium chloride, and 0.05% lysine (as L-Lysine hydrochloride).

2) The nutrient levels of the diets were analyzed.

SBM, soybean meal; GE, gross energy; CP, crude protein; EE, ether extract; NDF, neutral detergent fiber; ADF, acid detergent fiber; Ca, calcium; P, total phosphorus; Lys, lysine; Met, methionine.

**Table 4:** Composition and nutritional content of diets in Exp. 2 (%, as-fed basis)

Ingredient	Basal diet	SBM diets				

		2	3	4	5	6
Corn	90.00	76.50	76.50	76.50	76.50	76.50
SBM	7.00	5.95	5.95	5.95	5.95	5.95
Test SBM	-	14.55	14.55	14.55	14.55	14.55
Dicalcium phosphate	1.30	1.30	1.30	1.30	1.30	1.30
Salt	0.80	0.80	0.80	0.80	0.80	0.80
Limestone	0.40	0.40	0.40	0.40	0.40	0.40
Premix^1)^	0.50	0.50	0.50	0.50	0.50	0.50
Nutrient levels^2)^						
GE (MJ/kg)	15.65	15.86	15.88	15.92	16.00	15.88
CP	10.35	15.64	16.28	14.89	15.95	15.26
EE	1.49	1.81	1.67	1.76	1.33	1.42
NDF	8.26	9.20	9.37	9.52	8.71	9.06
ADF	1.87	2.32	2.48	2.84	2.52	2.48
Ash	3.42	4.11	4.26	4.19	4.38	4.06
Ca	0.63	0.57	0.60	0.61	0.59	0.63
P	0.44	0.46	0.48	0.54	0.45	0.49
Lys	0.45	0.76	0.74	0.74	0.76	0.73
Met	0.19	0.23	0.22	0.22	0.24	0.23

1) Premix provided per kilogram of complete feed: 6,000 IU of vitamin A (as retinyl acetatel), 3,000 IU of vitamin D_3_ (as cholecalciferol), 20 IU of vitamin E (as dl-alpha-tocopheryl acetate), 1.8 mg of vitamin K_3_ (as menadione nicotinamide bisulfite), 2.0 mg of vitamin B_1_ (as thiamine), 6.0 mg of vitamin B_2_ (as riboflavin), 4.0 mg of vitamin B_6_ (as pyridoxine hydrochloride), 3,000 mg of choline, 0.02 mg of vitamin B_12_ (as cobalamin), 26.0 mg of niacin, 18.0 mg of pantothenic acid (as dl-calcium pantothenate), 3.2 mg of folic acid, 0.4 mg of biotin, 400 mg of Fe (as FeSO_4_·H_2_O), 20 mg of Cu (as CuSO_4_·5H_2_O), 100 mg of Zn (as ZnO), 50 mg of Mn (as MnO), 1.2 mg of I (as KI), 0.30 mg of Se (as Na_2_SeO_3_), 8.0 g of Ca (as calcium pantothenate), 0.8 g of P (as calcium hydrogen phosphate), 5.6 g of sodium chloride, and 0.05% lysine (as L-Lysine hydrochloride).

2) The nutrient levels of the diets were analyzed.

SBM, soybean meal; GE, gross energy; CP, crude protein; EE, ether extract; NDF, neutral detergent fiber; ADF, acid detergent fiber Ca, calcium; P, total phosphorus; Lys, lysine; Met, methionine.

**Table 5:** Nutrients digestibility, energy utilization and nitrogen balance of experimental diets in Exp. 1^1)^

Item	Basal diet	Test diet 15	Test diet 30	SEM	p-value	

					Linear	Quadratic
ATTD (%)						
DM	90.1	89.9	89.2	0.23	0.272	0.559
GE	89.8	89.8	89.4	0.23	0.792	0.670
CP	85.2	88.9	89.6	0.55	<0.001	0.040
OM	92.4	92.3	91.6	0.20	0.267	0.473
Energy utilization (%)						
UE/DE	3.3	3.9	5.3	0.29	0.001	0.344
CH4E/DE	0.6	0.9	0.7	0.10	0.639	0.371
ME/DE	96.1	95.2	94.0	0.31	0.004	0.708
Nitrogen balance (g/day)						
N Intake	43.0	63.5	81.3	4.40	<0.001	0.347
Fecal N output	6.3	7.2	8.4	0.34	0.012	0.805
Urinary N output	20.0	26.7	45.5	3.41	<0.001	0.140
Retained N	16.8	29.5	27.4	2.27	0.040	0.104
N net utilization (%)	38.9	46.0	33.7	2.99	0.274	0.165

1) Data are least square means of 6 observations per treatment.

SEM, standard error of the mean; ATTD, apparent total tract digestibility; DM, dry matter; GE, gross energy; CP, crude protein; OM, organic matter; UE, urinary energy; DE, digestible energy; CH_4_E, methane energy; ME, metabolizable energy.

**Table 6:** Nutrients digestibility, energy utilization and nitrogen balance of experimental diets in Exp. 2^1)^

Item	Basal diet	SBM diets					SEM	p-value

		2	3	4	5	6		
ATTD (g/kg)								
DM	92.0^a^	90.1^ab^	89.4^b^	89.1^b^	90.9^ab^	88.9^b^	0.33	0.037
GE	91.4	89.8	89.1	89.1	90.6	88.6	0.33	0.121
CP	87.5	87.6	88.7	87.6	89.7	87.4	0.40	0.454
OM	93.8^a^	92.4^ab^	91.7^b^	91.5^b^	92.9^ab^	91.3^b^	0.26	0.031
Energy utilization (%)								
UE/DE	3.0	4.2	3.6	3.7	3.3	3.9	0.26	0.869
CH_4_E/DE	0.8	0.7	1.1	1.0	1.2	0.9	0.21	0.379
ME/DE	96.2	95.1	95.3	95.3	95.5	95.2	0.23	0.878
Nitrogen balance (g/day)								
N Intake	39.5^b^	58.9^a^	59.2^a^	55.2^a^	58.7^a^	56.5^a^	1.46	<0.001
Fecal N output	5.0	6.8	6.5	6.6	5.7	7.0	0.28	0.246
Urinary N output	10.1^y^	16.7^y^	25.1^x^	15.6^y^	16.2^y^	12.2^y^	1.35	0.074
Retained N	24.4^y^	35.4^x^	27.6^y^	33.0^x^	36.9^x^	37.3^x^	1.50	0.053
N net utilization (%)	60.9	60.3	46.6	59.7	62.9	66.4	2.33	0.425

1) Data are least square means of 6 observations per treatment.

a,b Means in the same row with no common superscript exhibit significant differences (p<0.05).

x,y Means in the same row with no common superscript exhibit a tendency of difference (p<0.10).

SBM, soybean meal; SEM, standard error of the mean; ATTD, apparent total tract digestibility; DM, dry matter; GE, gross energy; CP, crude protein; OM, organic matter; UE, urinary energy; DE, digestible energy; CH_4_E, methane energy; ME, metabolizable energy.

**Table 7:** Effects of dietary treatments on energy balance of pregnant sows and energy values of diets in Exp. 1^1)^

Item	Basal diet	Test diet 15	Test diet 30	SEM	p-value	

					Linear	Quadratic
Energy balance (kJ/kg BW^0.75^/day)						
ME intake	614.6	601.8	596.5	2.69	0.004	0.381
THP	494.2	465.6	479.1	5.33	0.109	0.075
FHP	381.0	349.6	356.4	4.80	0.010	0.033
RE (kJ/kg BW^0.75^/day)						
RE_P_	43.5	76.0	72.2	5.72	0.028	0.106
RE_L_	76.9	60.2	45.2	7.15	0.106	0.958
RE	120.4	136.2	117.4	4.46	0.225	0.097
Respiratory quotient						
Fed state	1.10	1.08	1.07	0.01	0.505	0.718
Fasted state	0.87	0.86	0.89	0.01	0.645	0.467
Energy value (MJ/kg DM)						
DE	16.34	16.42	16.44	0.05	0.704	0.839
ME	15.71	15.64	15.46	0.05	0.066	0.564
NE	12.82	12.63	12.28	0.11	0.074	0.716

1) Data are least square means of 6 observations per treatment.

SEM, standard error of the mean; BW, body weight; ME, metabolizable energy; THP, total heat production; FHP, fasting heat production; RE, retained of dietary energy; RE_P_, retained energy as protein; RE_L_, retained energy as lipid; DM, dry matter; DE, digestible energy; NE, net energy.

**Table 8:** Effects of dietary treatments on energy balance of pregnant sows and energy values of diets in Exp. 2^1)^

Item	Basal diet	SBM diets					SEM	p-value

		2	3	4	5	6		
Energy balance (kJ/kg BW^0.75^/day)								
ME intake	569.4	557.4	572.2	553.5	574.3	545.8	3.70	0.157
THP	448.1	427.2	453.9	461.5	465.4	457.0	10.02	0.919
FHP	342.4	324.7	344.2	355.2	350.4	352.0	8.78	0.935
RE (kJ/kg BW^0.75^/day)								
RE_P_	62.5^b^	92.8^a^	75.5^ab^	86.8^a^	99.6^a^	98.9^a^	3.92	0.031
RE_L_	58.9	37.4	42.8	5.3	9.3	−10.1	12.23	0.584
RE	121.3	130.2	118.3	92.0	109.0	88.8	10.89	0.861
Respiratory quotient								
Fed state	1.01	0.99	1.00	1.00	1.01	1.01	0.01	0.765
Fasted state	0.81	0.80	0.81	0.80	0.83	0.82	0.01	0.611
Energy value (MJ/kg DM)								
DE	15.92	16.00	16.13	15.96	16.22	15.86	0.06	0.700
ME	15.32	15.22	15.38	15.20	15.49	15.11	0.07	0.759
NE	12.46	12.42	12.43	12.28	12.40	12.20	0.13	0.994

1) Data are least square means of 6 observations per treatment.

a,b Means in the same row with no common superscript significantly differ (p<0.05).

SBM, soybean meal; SEM, standard error of the mean; BW, body weight; ME, metabolizable energy; THP, total heat production; FHP, fasting heat production; RE, retained of dietary energy; RE_P_, retained energy as protein; RE_L_, retained energy as lipid; DM, dry matter; DE, digestible energy; NE, net energy.

**Table 9:** Effect of substitution levels of SBM on the determination of energy values and energy utilization of soybean meal in Exp. 1^1)^

Item	Substitution level of 15%	Substitution level of 30%	SEM	p-value
Energy values (MJ/kg DM)				
DE	17.4	17.2	0.21	0.723
ME	15.7	15.3	0.31	0.584
NE	11.9	11.4	0.46	0.602
Energy utilization (%)				
ME/DE	90.4	89.3	1.49	0.723
NE/ME	75.8	74.1	2.63	0.766

1) Data are least square means of 6 observations per treatment.

SEM, standard error of the mean; DM, dry matter; DE, digestible energy; ME, metabolizable energy; NE, net energy.

**Table 10:** Energy values and energy utilization of SBM fed to pregnant sows in Exp. 2^1)^

Item	SBM					SEM	p-value

	2	3	4	5	6		
Energy values (MJ/kg DM)							
DE	16.9	17.8	16.7	18.5	16.0	0.53	0.678
ME	15.0	16.2	15.0	16.9	14.3	0.60	0.716
NE	12.6	12.7	11.6	12.5	11.1	1.05	0.990
Energy utilization (%)							
ME/D E	88.8	90.6	90.0	91.3	88.9	1.75	0.934
NE/ME	83.3	78.3	77.7	75.9	75.7	3.26	0.994

1) Data are least square means of 6 observations per treatment.

SBM, soybean meal; SEM, standard error of the mean; DM, dry matter; DE, digestible energy; ME, metabolizable energy; NE, net energy.

**Table 11:** Correlation coefficients between net energy and chemical composition of soybean meal

Item	NE	DE	ME	GE	CP	EE	NDF	ADF
DE	0.78							
ME	0.73	0.98**						
GE	0.96**	0.71	0.62					
CP	0.03	−0.41	−0.46	0.07				
EE	0.58	0.50	0.55	0.45	−0.50			
NDF	−0.33	−0.02	−0.08	−0.15	−0.17	−0.64		
ADF	−0.56	−0.27	−0.25	−0.51	−0.08	−0.59	0.77	
Ash	0.77	0.38	0.30	0.87*	0.06	0.44	−0.07	−0.32

* Represents 2 factors that were significantly correlated with p<0.05,

** represents 2 factors that were significantly correlated with p<0.01.

NE, net energy; DE, digestible energy; ME, metabolizable energy; GE, gross energy; CP, crude protein; EE, ether extract; NDF, neutral detergent fiber; ADF, acid detergent fiber.

**Table 12:** Prediction equations for available energy values (MJ/kg) from chemical composition of soybean meal (% or MJ/kg dry matter) in pregnant sows

No.	Prediction equations^1)^	R^2^	RMSE	p-value
1	DE = −194.30+(12.16×GE)−(3.62×Ash)	0.71	0.37	0.016
2	ME = −2.90+(1.06×DE)	0.98	0.02	<0.001
3	NE = −91.71+(5.35×GE)−(0.030×NDF)	0.96	0.03	0.009

1) These prediction equations were established using 6 samples of soybean meal.

RMSE, root mean square error; DE, digestible energy; GE, gross energy; ME, metabolizable energy; NE, net energy; NDF, neutral detergent fiber.

## References

[1] National Research Council (NRC) (2012). Nutrient requirements of swine.

[2] Li Z, Wang X, Guo P (2015). Prediction of digestible and metabolisable energy in soybean meals produced from soybeans of different origins fed to growing pigs. Arch Anim Nutr.

[3] Zhou X, Zhang HL, Lu XW (2022). Applying meta-data of soybean grain in origin trace and quarantine inspection. Food Res Int.

[4] Li Z, Lyu Z, Liu H (2021). Prediction of net energy values in expeller-pressed and solvent-extracted rapeseed meal for growing pigs. Anim Biosci.

[5] Wang K, Zou X, Guo L (2022). The nutritive value of soybean meal from different sources for sows during mid- and late gestation. J Anim Sci.

[6] Solà-Oriol D, Gasa J (2017). Feeding strategies in pig production: sows and their piglets. Anim Feed Sci Technol.

[7] Zhang S, Li Z, Liu L (2020). Concerns about the misleading conclusions regarding estimation of the net energy value of soybean meal relative to corn using the caloric efficiency approach in pigs. J Anim Sci Biotechnol.

[8] Li Z, Li Y, Lv Z (2017). Net energy of corn, soybean meal and rapeseed meal in growing pigs. J Anim Sci Biotechnol.

[9] Noblet J, Wu SB, Choct M (2022). Methodologies for energy evaluation of pig and poultry feeds: a review. Anim Nutr.

[10] Adeola O, Lewis DJ, Southern LL (2001). Digestion and balance techniques in pigs. Swine nutrition.

[11] Kim JW, Koo B, Nyachoti CM (2018). Net energy content of canola meal fed to growing pigs and effect of experimental methodology on energy values. J Anim Sci.

[12] He C, Xu S, Li Z (2024). Determination and prediction of the net energy content of wheat bran for pregnant sow. Arch Anim Nutr.

[13] Wang Z, Chen Y, Ding J (2019). Net energy content of five fiber-rich ingredients fed to pregnant sows. Anim Sci J.

[14] Casas GA, Stein HH (2017). Gestating sows have greater digestibility of energy in full fat rice bran and defatted rice bran than growing gilts regardless of level of feed intake. J Anim Sci.

[15] Gerrits W, Labussière E, Dijkstra J (2018). Recovery test results as a prerequisite for publication of gaseous exchange measurements. Animal.

[16] Association of Official Agricultural Chemists (AOAC) (2016). International Official methods of analysis of AOAC International.

[17] Johansen HN, Glitsø V, Bach Knudsen KE (1996). Influence of extraction solvent and temperature on the quantitative determination of oligosaccharides from plant materials by high-performance liquid chromatography. J Agric Food Chem.

[18] Van Soest PJ, Robertson JB, Lewis BA (1991). Methods for dietary fiber, neutral detergent fiber, and nonstarch polysaccharides in relation to animal nutrition. J Dairy Sci.

[19] Brouwer E, Blaxter K Report of sub-committee on constants and factors.

[20] Noblet J, Fortune H, Shi XS, Dubois S (1994). Prediction of net energy value of feeds for growing pigs. J Anim Sci.

[21] Labussiere E, Maxin G, Dubois S, van Milgen J, Bertrand G, Noblet J (2009). Effect of feed intake on heat production and protein and fat deposition in milk-fed veal calves. Animal.

[22] Grieshop CM, Kadzere CT, Clapper GM (2003). Chemical and nutritional characteristics of United States soybeans and soybean meals. J Agric Food Chem.

[23] Villamide MJ (1996). Methods of energy evaluation of feed ingredients for rabbits and their accuracy. Anim Feed Sci Technol.

[24] Le Bellego L, van Milgen J, Dubois S, Noblet J (2001). Energy utilization of low-protein diets in growing pigs. J Anim Sci.

[25] Le Goff G, Noblet J (2001). Comparative total tract digestibility of dietary energy and nutrients in growing pigs and adult sows. J Anim Sci.

[26] Kim H, Sung JY, Kim BG (2022). The influence of protein concentrations in basal diet on metabolizable energy of full-fat soybeans and soy protein isolate determined by the difference procedure in pigs. Anim Feed Sci Technol.

[27] Noblet J, Shi XS (1993). Comparative digestibility of energy and nutrients in growing pigs fed ad libitum and adults sows fed at maintenance. Livest Prod Sci.

[28] Le Goff G, Le Groumellec L, van Milgen J, Dubois S, Noblet J (2002). Digestibility and metabolic utilisation of dietary energy in adult sows: Influence of addition and origin of dietary fibre. Br J Nutr.

[29] Pérez de Nanclares M, Marcussen C, Tauson AH (2019). Increasing levels of rapeseed expeller meal in diets for pigs: effects on protein and energy metabolism. Animal.

[30] Fuller MF, Reeds PJ (1998). Nitrogen cycling in the gut. Annu Rev Nutr.

[31] Ramonet Y, van Milgen J, Dourmad JY, Dubois S, Meunier-Salauün MC, Noblet J (2000). The effect of dietary fibre on energy utilisation and partitioning of heat production over pregnancy in sows. Br J Nutr.

[32] Dahl WJ, Stewart ML (2015). Position of the academy of nutrition and dietetics: health implications of dietary fiber. J Acad Nutr Diet.

[33] Beyer M, Jentsch W, Hoffmann L, Schiemann R, Klein M (1994). Studies on energy and nitrogen metabolism of pregnant and lactating sows and suckling piglets: 4. chemical composition and energy content of the conception products, the reproductive organs as well as the live weight gains or losses of pregnant and lactating sows. Arch Tierernahr.

[34] Thomas LL, Goodband RD, Tokach MD, Dritz SS, Woodworth JC, DeRouchey JM (2018). Partitioning components of maternal growth to determine efficiency of feed use in gestating sows. J Anim Sci.

[35] Shimoda T, Hori S, Maegawa K (2020). A low coefficient of variation in hepatic triglyceride concentration in an inbred rat strain. Lipids Health Dis.

[36] Huang Q, Piao X, Liu L, Li D (2013). Effects of inclusion level on nutrient digestibility and energy content of wheat middlings and soya bean meal for growing pigs. Arch Anim Nutr.

[37] Navarro DMDL, Abelilla JJ, Stein HH (2019). Structures and characteristics of carbohydrates in diets fed to pigs: a review. J Anim Sci Biotechnol.

[38] Święch E (2017). Alternative prediction methods of protein and energy evaluation of pig feeds. J Anim Sci Biotechnol.

[39] Noblet J, Perez JM (1993). Prediction of digestibility of nutrients and energy values of pig diets from chemical analysis. J Anim Sci.

[40] Li DF, Qiao SY, Chen DW (2020). Nutrient requirements of swine in China.

